# Trends in disability in activities of daily living and instrumental activities of daily living among Chinese older adults from 2011 to 2018

**DOI:** 10.1007/s40520-023-02690-7

**Published:** 2024-02-07

**Authors:** Hua Ding, Kun Wang, Yanan Li, Xinyi Zhao

**Affiliations:** 1 Institute of Social Science Survey, Peking University, Beijing, China; 2 School of Health Humanities, Peking University, Beijing, China

**Keywords:** Activities of daily living, Disability, Instrumental activities of daily living, Trends

## Abstract

**Aim:**

This study aimed to assess the trends in disabilities in activities of daily living (ADL) and instrumental activities of daily living (IADL) among older Chinese adults and explore the influence of multimorbidity and unhealthy behaviors on ADL/IADL disability over time.

**Methods:**

Data were obtained from four waves (2011–2018) of the China Health and Retirement Longitudinal Study. Disability in ADL/IADL was defined as inability to perform any ADL/IADL task. Latent class analysis was used to identify multimorbidity patterns. The generalized estimating equation was used to test disability trends. Logistic regression was used to investigate the factors influencing disability.

**Results:**

The prevalence of IADL and ADL disability showed significant increasing trends among older Chinese adults from 2011 to 2018 (*p*_*trend*_ < 0.001). The negative association between alcohol intake more than once per month and IADL disability strengthened over time (*p*_*trend*_ < 0.05). The influence of the “arthritis/digestive diseases” pattern, “cardiometabolic disease” pattern and “high multimorbidity” pattern on ADL disability weakened over time (*p*_*trend*_ < 0.05).

**Conclusions:**

The prevalence of IADL and ADL disability among Chinese older adults increased over time. The “arthritis/digestive diseases” pattern, “cardiometabolic disease” pattern and “high multimorbidity” pattern appeared to be less disabling in ADL over time. Improving the prevention and treatment of multimorbidity and developing age-friendly living conditions could be helpful to reduce the risks of disability.

**Supplementary Information:**

The online version contains supplementary material available at 10.1007/s40520-023-02690-7.

## Introduction

Aging is accompanied by deterioration of physical functioning [1], which increases the prevalence of disability in activities of daily living (ADL) and instrumental activities of daily living (IADL) of older adults, further increasing the demand for long-term care [2]. The trend of disability, which could predict the needs of long-term care, has drawn increasing attention from scholars. China, a country with the largest older population (i.e., 264 million people aged 60 and above [3]) in the world, has been facing tremendous challenges in caring for older adults.

The findings of numerous empirical studies on the trend of disability in Western societies were inconsistent. Several studies showed a declining trend of disability among American people aged 65 and older from the late twentieth century to the early twenty-first century [4, 5]. In contrast, another study revealed that the prevalence of disability increased or remained stable among Americans aged 53–88 years from 1996 to 2010 [6]. Similarly, studies in European countries showed different trends in disability among older adults [7–10]. In terms of studies on disability trends in China, some used data that were not representative of the total Chinese population [11], some used data collected prior to 2010 [1, 11, 12], and some focused only on ADL disability [1, 2, 12, 13]. In the past decade, China has undergone dramatic social and economic transformations, which may have negative or positive impacts on the health of older adults [14]. Moreover, IADL is essential for maintaining household living and community interaction [15], and IADL disability usually precedes ADL disability [16]. Therefore, there is a need for up-to-date research on disability trends, and both ADL and IADL should be used to measure older people’s dependency [17, 18].

Earlier studies showed that functional disability was associated with demographic characteristics (such as age, gender, educational level, and residence) [19], unhealthy behaviors [20] and chronic conditions [21, 22]. In terms of research on the association between chronic conditions and functional disability, the classical single-disease paradigm might not be adequate, and simplified binary multimorbidity variable might obscure the different impacts among diseases [23]. Zhou and colleagues derived four patterns from 13 chronic diseases to demonstrate the impact of multimorbidity on the incidence of disability [24]. Liu and colleagues yielded five patterns from 14 diseases and demonstrated their relationship with physical limitations [25]. However, studies on the impact of multimorbidity patterns on trends in ADL and IADL disability are still limited.

Hence, this study aimed to enhance the understanding of ADL and IADL disability trends among older Chinese adults using nationally representative data. Moreover, we aimed to explore the dynamic effects of unhealthy behaviors and multimorbidity patterns on ADL and IADL disability over time.

## Methods

### Data and sampling

This study used data from four waves of the China Health and Retirement Longitudinal Study (CHARLS). The 2011 baseline survey was conducted in 28 provinces, 150 counties/districts, and 450 villages/urban communities across the country and involved 17,708 individuals aged 45 and older. It was followed by wave 2 in 2013, wave 3 in 2015, and wave 4 in 2018 [26]. Information was collected by trained interviewers through face-to-face computer-assisted personal interview (CAPI), which covered sociodemographics, family structure, health status and functioning, health care and insurance, and income and consumption. This study focused on individuals who were 60 years or older and had information on ADLs/IADLs in either wave of the CHARLS, including 7510 participants in 2011, 8677 participants in 2013, 10,062 participants in 2015, and 10,975 participants in 2018; these participants included those lost to follow-up and those who died. The flowchart of sample selection is shown in Supplementary Fig. 1.

### Measurement

#### ADL or IADL difficulty

CHARLS asked participants whether they had limitations in six ADL tasks (dressing, bathing, eating, indoor transferring, going to the toilet, and continence) and five IADL tasks (doing household chores, cooking, shopping, wealth management, and taking medications). Each question had four options: “I do not have any difficulty,” “I have difficulty but can do it,” “I need help,” and “I cannot do it.” Based on the Katz index scale [27] and previous research [28], participants who chose the last two options in their responses to any of the six ADL tasks or five IADL tasks were considered to have ADL or IADL disability.

### Control variables

The sociodemographic variables included age, gender, residence (urban/rural), education (no formal school/elementary school/middle school or above) and marital status (married/unmarried). Unhealthy behaviors included consuming alcohol more than once per month in the past year (yes/no) and ever smoking (yes/no). Multimorbidity was defined by having at least two chronic diseases among the 14 conditions (i.e., hypertension, dyslipidemia, diabetes, cancer, heart disease, lung disease, liver disease, stroke, kidney disease, digestive disease, emotional problems, memory-related disease, arthritis, and asthma) according to the CHARLS questionnaire. Latent class analysis (LCA) was utilized to identify the patterns of multimorbidity (see Statistical Analysis).

### Statistical analysis

Trends in the prevalence of ADL and IADL disability were assessed using the generalized estimating equation (GEE) with logistic links and binomial distributions to accommodate the correlation of measurements within individuals across waves. The odds ratios (ORs) and confidence intervals (CIs) for ADL disability and IADL disability in 2013, 2015, and 2018 relative to 2011 were estimated. The associations of ADL and IADL disability with unhealthy behaviors and multimorbidity in each wave were examined through logistic regression. The secular trend in the magnitude of the associations between disability and the influencing factors was examined using the interaction term in GEE models. Multivariate analyses were unweighted to adjust for covariates in all models. LCA was used to identify the patterns of multimorbidity based on chronic disease information first provided by participants. A variety of model fit statistics and clinical meanings were taken into consideration when choosing the best fitting solution. We also conducted a sensitivity analysis by repeating the GEE model using samples who had participated in all four waves of the CHARLS. LCA was conducted using Mplus 8.3. Other analyses were performed using SAS software 9.4.

## Results

### Characteristics of participants

Table 1 presents the characteristics of the participants in the four waves. The mean age of the participants increased from 68.5 (SD = 7.1) in 2011 to 68.4 (SD = 7.0) in 2018. Most of the subjects in each wave lived in rural areas (75.1–76.2%) and were married (78.1–78.8%). More than half of the participants did not receive formal education (51.5–57.1%). The proportion of participants who drank alcohol more than once a month in the past year increased from 2011 to 2015, as did the proportion of participants who smoked. The proportion of participants with chronic conditions decreased from 74.3% in 2011 to 63.1% in 2018. The proportions of subjects in the “respiratory diseases” pattern, “cardiometabolic diseases” pattern and “arthritis/digestive diseases” pattern decreased, while the proportion in the “high multimorbidity” pattern remained almost unchanged (details of multimorbidity patterns are reported in the “Multimorbidity Patterns” section).

**Table 1:** Characteristics of the participants in 2011, 2013, 2015, and 2018

Characteristics	2011 (n = 7510)	2013 (n = 8677)	2015 (n = 10,062)	2018 (n = 10,975)
Age, mean (SD)	68.4 (7.0)	68.5 (7.1)	68.7 (7.1)	69.4 (7.3)
Age (years), n (%)				
60–69	4652 (61.9)	5399 (62.2)	6249 (62.1)	6472 (59.0)
70–79	2214 (29.5)	2495 (28.8)	2886 (28.7)	3308 (30.1)
≥ 80	644 (8.6)	783 (9.0)	927 (9.2)	1195 (10.9)
Gender, n (%)				
Male	3768 (50.2)	4328 (49.9)	4949 (49.2)	5362 (48.9)
Female	3742 (49.8)	4349 (50.1)	5113 (50.8)	5613 (51.1)
Residence, n (%)				
Rural	5678 (75.7)	6516 (75.1)	7483 (76.2)	8350 (76.1)
Urban	1827 (24.3)	2159 (24.9)	2339 (23.8)	2624 (23.9)
Education, n (%)				
No formal school	4286 (57.1)	4886 (56.3)	5415 (55.3)	5652 (51.5)
Elementary school	1774 (23.6)	2010 (23.2)	2214 (22.6)	2403 (21.9)
Middle school or above	1446 (19.3)	1779 (20.5)	2160 (22.1)	2920 (26.6)
Marital status, n (%)				
Married	5865 (78.1)	6839 (78.8)	7903 (78.5)	8566 (78.1)
Unmarried	1645 (21.9)	1838 (21.2)	2159 (21.5)	2409 (22.0)
Alcohol intake (> once/month), n (%)	1746 (23.3)	2111 (24.4)	2455 (24.4)	2648 (24.1)
Ever smoking, n (%)	3162 (42.1)	3955 (45.6)	4698 (46.7)	4964 (45.2)
Multimorbidity pattern, n (%)				
No chronic diseases	1851 (25.7)	2425 (29.3)	3124 (33.9)	3803 (36.9)
Arthritis/ digestive diseases	2345 (32.5)	2548 (30.8)	2680 (29.0)	2971 (28.9)
Respiratory diseases	707 (9.8)	729 (8.8)	706 (7.7)	676 (6.6)
Cardiometabolic diseases	2001 (27.7)	2219 (26.8)	2327 (25.2)	2409 (23.4)
High multimorbidity	309 (4.3)	355 (4.3)	391 (4.2)	439 (4.3)
Multimorbidity, n (%)				
No	3938 (53.3)	4024 (47.8)	4202 (48.1)	5798 (52.8)
Yes	3446 (46.7)	4389 (52.2)	4526 (51.9)	5177 (47.2)

The number of subjects with missing values was 248 for residence, 279 for education, 19 for alcohol intake, 5 for ever smoking, 2209 for multimorbidity pattern and 1724 for multimorbidity

### Prevalence of ADL and IADL disability

**Fig. 1:**
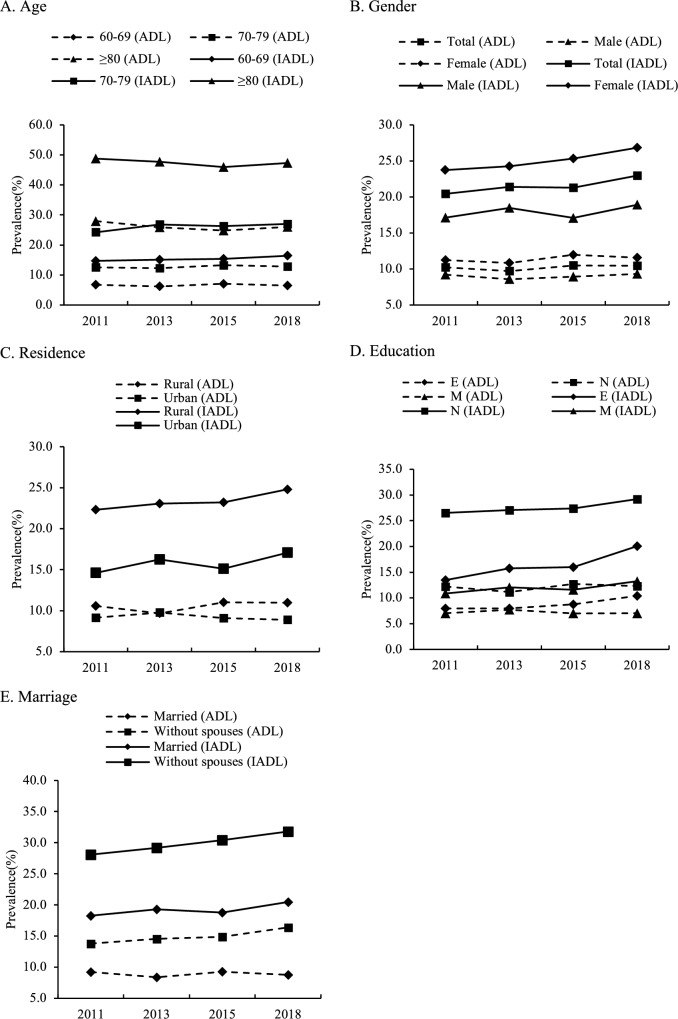
The weighted prevalence of ADL/IADL disability in 2011, 2013, 2015 and 2018 by demographic groups. In graph D, *E* elementary school, *N* no formal school, *M* middle school or above

Figure 1 demonstrates the weighted prevalence of ADL and IADL disability in total and as grouped by age, gender, residence, education, and marital status. The prevalence of ADL disability changed from 10.2% in 2011 to 10.5% in 2018, while the prevalence of IADL disability increased from 20.4% in 2011 to 23.0% in 2018. At each wave, the prevalence of IADL disability was nearly or greater than twice that of ADL disability. Participants with older ages had a greater incidence of disability (Fig. 1, Graph A). The trend in the prevalence of ADL or IADL disability for participants of different genders was similar to that for the overall participants, and females were more likely to have ADL or IADL disability (Fig. 1, Graph B). Participants who were living in rural areas (Fig. 1, Graph C), less educated (Fig. 1, Graph D), or unmarried (Fig. 1, Graph E) had a greater prevalence of ADL or IADL disability.

### Trends in prevalence of ADL and IADL disability

Table 2 presents trends in ADL or IADL disability from 2011 to 2018 after controlling for age, gender, residence, education, marital status, smoking status, alcohol intake, and multimorbidity. There was an upward trend in the prevalence of ADL disability in the total sample (*p*_*trend*_ < 0.001). Subgroup analysis revealed similar increasing trends within participants in almost all subgroups (*p*_*trend*_ < 0.05), except for those aged 80 or above, those living in urban areas, those with higher education levels, and those drinking alcohol more than once per month in the past year (*p*_*trend*_ > 0.05). Table 2 also shows that compared with the odds ratios of IADL disability prevalence for the total sample in 2011, the odds ratios were 9% higher in 2013, 13% higher in 2015, and 36% higher in 2018, showing an increasing trend (*p*_*trend*_ < 0.001). There were also upward trends in IADL disability over time in all subgroups (*p*_*trend*_ < 0.05), only except for participants who drank more than once per month in the past year (*p*_*trend*_ > 0.05).

**Table 2:** Trends in the prevalence of ADL or IADL disability for participants aged 60 and over, 2011–2018

Variables		2011		2013		2015		2018		Ptrend^†^
		n/N	Ref	n/N	OR (95% CI)	n/N	OR (95% CI)	n/N	OR (95% CI)	
Total	ADL	768/7510	1.00	842/8677	0.95 (0.86–1.04)	1055/10062	1.12 (1.01–1.23)	1148/10975	1.17 (1.06–1.29)	< .001
	IADL	1534/7510	1.00	1855/8677	1.09 (1.02–1.17)	2140/10062	1.13 (1.05–1.22)	2520/10975	1.36 (1.26–1.46)	< .001
Age, years										
60–69	ADL	311/4652	1.00	333/5399	0.96 (0.82–1.12)	442/6249	1.20 (1.03–1.39)	415/6472	1.21 (1.04–1.41)	.001
	IADL	684/4652	1.00	814/5399	1.03 (0.93–1.15)	957/6249	1.11 (1.00–1.24)	1061/6472	1.35 (1.21–1.50)	< .001
70–79	ADL	277/2214	1.00	306/2495	1.00 (0.84–1.18)	383/2886	1.19 (1.01–1.41)	422/3308	1.26 (1.06–1.50)	.001
	IADL	536/2214	1.00	688/2495	1.24 (1.10–1.41)	757/2886	1.31 (1.15–1.50)	893/3308	1.57 (1.37–1.78)	< .001
≥ 80	ADL	180/644	1.00	203/783	0.91 (0.73–1.14)	230/927	1.01 (0.80–1.28)	311/1195	1.08 (0.86–1.36)	.257
	IADL	314/644	1.00	373/783	1.08 (0.90–1.30)	426/927	1.03 (0.84–1.26)	566/1195	1.31 (1.07–1.59)	.008
Gender										
Male	ADL	347/3768	1.00	371/4328	0.94 (0.81–1.08)	443/4949	1.04 (0.90–1.20)	499/5362	1.19 (1.03–1.37)	.003
	IADL	646/3768	1.00	800/4328	1.14 (1.02–1.27)	846/4949	1.08 (0.96–1.21)	1014/5362	1.36 (1.22–1.52)	< .001
Female	ADL	421/3742	1.00	471/4349	0.96 (0.84–1.10)	612/5113	1.19 (1.04–1.36)	649/5613	1.16 (1.02–1.33)	.004
	IADL	888/3742	1.00	1055/4349	1.05 (0.96–1.16)	1294/5113	1.17 (1.06–1.29)	1506/5613	1.36 (1.23–1.50)	< .001
Residence										
Rural	ADL	601/5678	1.00	630/6516	0.91 (0.81–1.02)	825/7483	1.11 (0.99–1.24)	915/8350	1.16 (1.04–1.30)	< .001
	IADL	1267/5678	1.00	1503/6516	1.07 (0.99–1.16)	1737/7483	1.11 (1.02–1.20)	2071/8350	1.32 (1.22–1.43)	< .001
Urban	ADL	167/1827	1.00	211/2159	1.09 (0.90–1.32)	213/2339	1.11 (0.90–1.37)	233/2624	1.14 (0.93–1.40)	.237
	IADL	267/1827	1.00	351/2159	1.20 (1.02–1.41)	354/2339	1.24 (1.05–1.47)	448/2624	1.50 (1.27–1.77)	< .001
Education										
No formal school	ADL	524/4286	1.00	545/4886	0.93 (0.82–1.05)	689/5415	1.16 (1.03–1.31)	693/5652	1.15 (1.02–1.30)	.001
	IADL	1137/4286	1.00	1324/4886	1.09 (1.00–1.18)	1485/5415	1.13 (1.03–1.24)	1651/5652	1.31 (1.20–1.44)	< .001
Elementary school	ADL	141/1774	1.00	160/2010	0.91 (0.72–1.15)	194/2214	1.05 (0.83–1.32)	250/2403	1.26 (1.01–1.58)	.006
	IADL	239/1774	1.00	316/2010	1.11 (0.94–1.31)	354/2214	1.20 (1.01–1.43)	482/2403	1.50 (1.26–1.79)	< .001
≥ Middle school	ADL	102/1446	1.00	137/1779	1.05 (0.84–1.32)	151/ 2160	0.98 (0.77–1.24)	205/2920	1.04 (0.82–1.33)	.831
	IADL	157/1446	1.00	215/1779	1.07 (0.88–1.31)	250/2160	1.04 (0.85–1.27)	387/2920	1.28 (1.05–1.55)	.009
Marriage										
Married	ADL	541/5865	1.00	574/6839	0.93 (0.83–1.04)	733/7903	1.13 (1.00–1.27)	753/8566	1.14 (1.02–1.29)	.001
	IADL	1072/5865	1.00	1319/6839	1.09 (1.00–1.19)	1483/7903	1.11 (1.02–1.21)	1754/8566	1.36 (1.25–1.48)	< .001
Unmarried	ADL	227/1645	1.00	268/1838	0.98 (0.82–1.18)	322/2159	1.06 (0.88–1.28)	395/2409	1.19 (0.99–1.43)	.026
	IADL	462/1645	1.00	536/1838	1.08 (0.94–1.24)	657/2159	1.16 (1.01–1.34)	766/2409	1.30 (1.13–1.50)	< .001
Alcohol intake										
> once/month	ADL	129/1746	1.00	100/2111	0.65 (0.50–0.85)	143/2455	0.83 (0.65–1.07)	139/2648	0.82 (0.64–1.06)	.574
	IADL	242/1746	1.00	277/2111	1.00 (0.83–1.20)	309/24655	0.97 (0.80–1.16)	335/2648	1.09 (0.91–1.30)	.305
≤ once/month or never	ADL	639/5762	1.00	739/6556	0.98 (0.88–1.09)	910/7600	1.16 (1.04–1.29)	1009/8327	1.21 (1.09–1.35)	< .001
	IADL	1291/5762	1.00	1574/6556	1.10 (1.01–1.19)	1829/7600	1.15 (1.06–1.25)	2185/8327	1.39 (1.28–1.51)	< .001
Smoking										
Ever smoking	ADL	308/3162	1.00	377/3955	0.96 (0.83–1.12)	473/4698	1.10 (0.95–1.27)	503/4964	1.19 (1.03–1.39)	.003
	IADL	602/3162	1.00	796/3955	1.09 (0.98–1.22)	905/4698	1.08 (0.97–1.22)	1027/4964	1.33 (1.18–1.49)	< .001
Never smoking	ADL	460/4347	1.00	465/4720	0.92 (4.81–1.15)	581/5362	1.11 (8.98–1.28)	645/6011	1.12 (4.99–1.28)	.013
	IADL	931/4347	1.00	1058/4720	1.07 (0.98–1.18)	1234/5362	1.15 (1.04–1.27)	1493/6011	1.36 (1.24–1.50)	< .001

Adjusting for age, gender, residence, education, marital status, ever smoking, alcohol intake, and multimorbidity

*ADL* activities of daily living, *IADL* instrumental activities of daily living, *OR* odds ratio, *CI* confidence interval

### Relative annual changes in the odds ratios of ADL and IADL disability

According to the univariate model (Table 3, Model 1), the odds ratios of ADL and IADL disability increased by 4.5% and 5.2%, respectively, per year. After controlling for a set of covariates, the relative average annual changes in the odds ratios of ADL and IADL disability were 2.8% and 4.3%, respectively (Table 3, Model 4). The annual changes in the odds ratio of ADL disability were significantly positive among all subgroups, except for those aged 80 years or over, living in urban areas and with education levels of middle school or above (Table 3, Model 4). Similarly, the annual changes in the odds ratios of IADL disability were significantly positive in all subgroups (Table 3, Model 4).

**Table 3:** Average annual changes at 95% Confidence Interval in the odds ratio of ADL/IADL disability, 2011–2018

Variables		Average Annual Changes (95% CI), %				P
		Model 1	Model 2	Model 3	Model 4	
Total	ADL	4.5 (3.3–5.7)	2.6 (1.4–3.8)	2.4 (1.2–3.6)	2.8 (1.5–4.1)	< .001
	IADL	5.2 (4.3–6.0)	3.7 (2.8–4.6)	3.6 (2.7–4.5)	4.3 (3.3–5.3)	< .001
Age (years)						
60–69	ADL	2.9 (1.0–4.8)	3.0 (1.1–4.9)	2.9 (1.0–4.8)	3.4 (1.3–5.5)	.001
	IADL	3.5 (2.2–4.8)	3.6 (2.3–5.0)	3.6 (2.2–5.0)	4.5 (3.0–6.0)	< .001
70–79	ADL	3.1 (1.1–5.2)	3.5 (1.4–5.6)	3.5 (1.4–5.6)	3.8 (1.5–6.1)	.001
	IADL	4.0 (2.5–5.6)	5.2 (3.5–6.8)	5.1 (3.5–6.8)	5.9 (4.1–7.6)	< .001
≥ 80	ADL	2.0 (− 0.8–4.9)	2.3 (− 0.6–5.1)	1.8 (− 1.1–4.7)	1.8 (− 1.3–4.9)	.257
	IADL	3.4 (0.9–5.8)	4.1 (1.5–6.6)	3.4 (0.8–6.0)	3.7 (1.0–6.5)	.008
Gender						
Male	ADL	4.7 (3.0–6.4)	2.6 (0.8–4.4)	2.3 (0.5–4.1)	3.0 (1.0–4.9)	.003
	IADL	4.8 (3.5–6.1)	3.3 (1.9–4.7)	3.0 (1.7–4.4)	4.1 (2.6–5.6)	< .001
Female	ADL	4.3 (2.7–5.9)	2.5 (0.9–4.2)	2.6 (0.9–4.2)	2.6 (0.9–4.4)	.004
	IADL	5.3 (4.2–6.5)	4.0 (2.8–5.3)	4.1 (2.9–5.3)	4.6 (3.2–5.9)	< .001
Residence						
Rural	ADL	4.7 (3.4–6.1)	2.9 (1.5–4.2)	2.7 (1.3–4.0)	3.0 (1.5–4.5)	< .001
	IADL	4.8 (3.8–5.8)	3.5 (2.5–4.5)	3.4 (2.4–4.4)	4.0 (2.9–5.1)	< .001
Urban	ADL	3.1 (0.7–5.5)	0.9 (− 1.6–3.4)	0.9 (− 1.6–3.5)	1.7 (− 1.1–4.4)	.210
	IADL	5.8 (3.9–7.7)	3.9 (1.9–5.9)	4.0 (2.0–6.0)	5.4 (3.1–7.6)	< .001
Education						
No formal school	ADL	4.2 (2.7–5.7)	2.7 (1.1–4.2)	2.5 (1.0–4.0)	2.7 (1.1–4.4)	.001
	IADL	4.6 (3.4–5.7)	3.1 (2.0–4.3)	3.1 (1.9–4.2)	3.8 (2.6–5.1)	< .001
Elementary school	ADL	7.6 (4.9–10.2)	3.9 (1.2–6.6)	3.5 (0.8–6.3)	4.2 (1.2–7.2)	.006
	IADL	8.8 (6.8–10.8)	5.5 (3.4–7.5)	5.2 (3.0–7.3)	5.9 (3.6–8.2)	< .001
≥ Middle school	ADL	2.7 (− 0.3–5.7)	− 0.5 (− 3.5–2.5)	− 0.4 (− 3.4–2.6)	0.4 (− 2.9–3.6)	.831
	IADL	5.9 (3.6–8.3)	2.6 (0.2–4.9)	2.7 (0.3–5.1)	3.4 (0.9–6.0)	.009
Marriage						
Married	ADL	3.7 (2.3–5.1)	1.9 (0.5–3.4)	1.8 (0.3–3.2)	2.6 (1.0–4.2)	.001
	IADL	4.7 (3.6–5.7)	3.4 (2.3–4.5)	3.3 (2.2–4.4)	4.4 (3.2–5.5)	< .001
Unmarried	ADL	5.2 (3.1–7.4)	3.7 (1.4–5.9)	3.5 (1.2–5.7)	2.8 (0.3–5.3)	.026
	IADL	5.2 (3.6–6.9)	3.8 (1.9–5.6)	3.7 (1.9–5.5)	3.7 (1.8–5.7)	< .001

Model 1 was univariate; Model 2 was controlled for age, gender, residence, education and marital status; Model 3 was additionally controlled for lifestyle factors (ever smoking and alcohol intake); and Model 4 was further controlled for multimorbidity

*CI* confidence interval

### Multimorbidity patterns

Results of LCA showed that the four-class model yielded an appropriate fit and had reasonable clinical results for interpretability (see Supplementary Table 1 and Supplementary Fig. 2). Approximately 45.8% of the participants were classified into the “arthritis/digestive diseases” pattern (i.e., participants in this pattern had a high prevalence of arthritis and digestive diseases), 11.4% were labeled the “respiratory diseases” pattern (i.e., participants in this pattern had a high prevalence of lung diseases and asthma), 34.8% were classified as the “cardiometabolic diseases” pattern (i.e., participants in this group showed a high prevalence of hypertension, dyslipidemia, diabetes, heart diseases and stroke), and 8.0% belonged to the “high multimorbidity” pattern (i.e., participants in this group reported a higher prevalence of most conditions than those in other groups).

### Association of ADL/IADL disability with unhealthy behaviors and chronic conditions

Logistic modeling was used to analyze the influence of unhealthy behaviors and multimorbidity on ADL or IADL disability in each wave, and GEE was subsequently used to investigate the trend of the associations over time. As shown in Table 4, participants who drank alcohol more than once per month were less likely to report ADL and IADL disability in each wave (OR < 1). The GEE showed that the negative association between alcohol intake and IADL disability strengthened over time (OR < 1, interaction effect < 0, and *p*_*trend*_ < 0.05). Participants who had smoked were more likely to report ADL and IADL disability in each wave (OR > 1). However, we did not observe an upward or downward trend in the relationship between smoking and ADL/IADL disability over time (*p*_*trend*_ > 0.05).

**Table 4:** Trends in the associations of unhealthy behaviors and chronic diseases and ADL/IADL disability, 2011–2018

Variables		2011 (n = 7626)		2013 (n = 8850)		2015 (n = 10,101)		2018 (n = 10,975)		Interaction effect	Ptrend^*^
		n/N	OR (95% CI)	n/N	OR (95% CI)	n/N	OR (95% CI)	n/N	OR (95% CI)		
Alcohol intake	ADL	129/1746	0.78 (0.62–0.98)	100/2111	0.48 (0.38–0.61)	143/2455	0.60 (0.48–0.74)	139/2648	0.50 (0.41–0.61)	− 0.030	0.110
	IADL	242/1746	0.67 (0.56–0.80)	277/2111	0.60 (0.51–0.70)	309/2455	0.63 (0.54–0.74)	335/2648	0.52 (0.45–0.60)	− 0.028	0.032
Ever smoking	ADL	308/3162	1.06 (0.86–1.30)	377/3955	1.24 (1.01–1.52)	473/4698	1.25 (1.02–1.54)	503/4964	1.24 (1.03–1.50)	0.009	0.524
	IADL	602/3162	1.17 (1.00–1.38)	796/3955	1.10 (0.95–1.28)	905/4698	1.20 (1.02–1.42)	1027/4964	1.17 (1.01–1.35)	− 0.005	0.646
Multimorbidity pattern											
No chronic diseases	ADL	71/1851	1.00 (ref)	140/2425	1.00 (ref)	189/3124	1.00 (ref)	241/3808	1.00 (ref)	–	–
	IADL	228/1851	1.00 (ref)	365/2425	1.00 (ref)	469/3124	1.00 (ref)	648/3808	1.00 (ref)	–	–
Arthritis/digestive	ADL	221/2345	1.89 (1.39–2.58)	205/2548	0.96 (0.74–1.25)	276/2680	1.31 (1.01–1.69)	301/2971	1.34 (1.09–1.64)	− 0.040	0.037
	IADL	437/2345	1.22 (0.99–1.49)	524/2548	1.16 (0.98–1.39)	560/2680	1.22 (1.02–1.46)	679/2971	1.17 (1.01–1.35)	− 0.022	0.107
Respiratory	ADL	79/707	1.74 (1.19–2.54)	92/729	1.17 (0.85–1.61)	95/706	1.46 (1.05–2.02)	109/676	1.80 (1.36–2.38)	0.003	0.914
	IADL	190/707	1.63 (1.26–2.12)	207/729	1.49 (1.18–1.87)	214/706	1.75 (1.37–2.22)	222/676	1.69 (1.37–2.08)	− 0.003	0.886
Cardiometabolic	ADL	277/2001	2.75 (2.02–3.75)	276/2219	1.37 (1.06–1.77)	302/2327	1.70 (1.32–2.19)	312/2409	1.71 (1.39–2.11)	− 0.056	0.004
	IADL	459/2001	1.61 (1.30–1.98)	530/2219	1.40 (1.17–1.68)	544/2327	1.48 (1.23–1.78)	616/2409	1.39 (1.19–1.62)	− 0.025	0.071
High multimorbidity	ADL	70/309	4.33 (2.85–6.56)	66/355	1.97 (1.36–2.84)	76/391	2.44 (1.71–3.49)	83/439	2.38 (1.74–3.27)	− 0.090	0.002
	IADL	116/309	2.91 (2.11–4.01)	110/355	1.84 (1.37–2.46)	138/391	2.51 (1.90–3.33)	168/439	2.24 (1.75–2.86)	− 0.038	0.100
Multimorbidity											
No	ADL	234/3938	1.00 (ref)	216/4024	1.00 (ref)	224/4202	1.00 (ref)	385/5798	1.00 (ref)	–	–
	IADL	561/3938	1.00 (ref)	604/4024	1.00 (ref)	581/4202	1.00 (ref)	991/5798	1.00 (ref)	–	–
Yes	ADL	519/3446	2.04 (1.67–2.49)	592/4389	2.46 (2.00–3.02)	673/4526	2.27 (1.85–2.78)	763/5177	1.74 (1.47–2.06)	− 0.045	0.001
	IADL	933/3446	1.89 (1.63–2.21)	1173/4389	1.84 (1.60–2.12)	1248/4526	1.83 (1.58–2.12)	1529/5177	1.60 (1.42–1.81)	− 0.021	0.043

Adjusting for age, gender, residence, education, marital status, ever smoking and alcohol intake in the table

*ADL* activities of daily living, *IADL* instrumental activities of daily living, *OR* odds ratio, *CI* confidence interval

As shown in Table 4, participants with multimorbidity were more likely to have ADL and IADL disability than those without multimorbidity in each wave (OR > 1). The GEE showed that the disabling effect of multimorbidity on ADL/IADL significantly weakened over time (OR > 1, interaction effect < 0, and *p*_*trend*_ < 0.05). Compared with those with no chronic conditions, participants in any of the four multimorbidity patterns were more likely to report ADL/IADL disability in almost all waves (OR > 1). The GEE model showed that the effects of the “arthritis/digestive diseases” pattern, “cardiometabolic diseases” pattern and “high morbidity” pattern on ADL disability significantly weakened over time (OR > 1, interaction effect < 0, and *p*_*trend*_ < 0.05).

### Results of the sensitivity analyses

We conducted sensitivity analyses by repeating the analytical models using samples from individuals who participated in all four waves. These results were similar to those of the main analysis. For instance, we found upward trends in the prevalence of ADL and IADL disability among the participants from 2011 to 2018 (*p*_*trend*_ < 0.001) (see Supplementary Table 2). After controlling for a set of covariates, the relative average annual changes in the odds ratios of ADL and IADL disability were positive (Supplementary Table 3, Model 4). The effects of multimorbidity and the “high multimorbidity” pattern on ADL disability mitigated significantly over time (OR > 1, interaction effect < 0, *p*_*trend*_ < 0.05) (see Supplementary Table 4).

## Discussion

There was an increasing trend in the prevalence of ADL and IADL disability among older Chinese adults from 2011 to 2018. The odds ratios of ADL and IADL disability also significantly increased each year. The negative association between alcohol intake and IADL disability strengthened over time. The deteriorating impact of multimorbidity on ADL and IADL, as well as the disabling effect of the “arthritis/digestive diseases” pattern, “cardiometabolic diseases” pattern and “high multimorbidity” pattern on ADL, weakened over time.

The proportion of older people with ADL disability was close to or above 10% in each wave in our study, which was consistent with the findings of previous research [29]. In addition, we found that the prevalence of IADL disability was nearly or greater than 20% per wave. Although older adults with ADL disability are more urgently in need of personal care and social services, as IADL disability is reflective of people’s capacity for instrumental activities in real-life situations and usually declines prior to ADL disability, healthcare staff and health policy makers should pay attention to the high prevalence of IADL disability and provide early intervention.

The prevalence of ADL and IADL disability increased from 2011 to 2018 in the present study. These findings were similar to those of studies in the United States [6] and Spain [30], which revealed an increase in the prevalence of disability; however, these findings were inconsistent with those of Feng and colleagues’ research in Shanghai, China, which reported a substantial improvement in ADL/IADL functioning among older people from 1998 to 2008 [11]. This might be because of the differences in disability measurements and because the data from Feng’s study [11] were collected in one of the most developed cities in China. Improvements in the living environment, health services, and rehabilitation medical technology in modern cities might mitigate functional impairments among older adults [31].

Our study showed that participants who were older, female, unmarried, less educated, and living in rural areas were more likely to have ADL or IADL disability, which was in line with previous findings [19, 28, 30, 31]. Older individuals tend to have lower levels of physical activity and poorer extremity performance, and thus having a greater risk of developing ADL and IADL disability. Evidence has shown that females have lower mortality and recovery rates than males, which contributes to a higher prevalence of disability in females [30]. Individuals with lower socioeconomic status (such as less educated and living in rural areas) might be less likely to receive good-quality medical and/or rehabilitation care, leading to higher risks of ADL/IADL.

Our study revealed that alcohol intake for more than once per month was negatively associated with IADL disability, and this association strengthened over time. Although excessive drinking was related to IADL impairments [32], evidence showed that moderate alcohol drinkers had a lower risk of IADL limitations [33]. In China, the proportion of excessive drinkers is very small. A study of middle-aged and older people in urban China showed that excessive drinkers accounted for only 4% of the population [34]. Modest alcohol consumption has been reported to be beneficial for cardiovascular health and cognitive function [35, 36], thus preventing IADL impairments.

We found that the impact of multimorbidity on ADL and IADL disability weakened over time, and the effects of the “arthritis/digestive diseases” pattern, “cardiometabolic diseases” pattern and “high multimorbidity patterns” on ADL disability mitigated over time. Previous studies showed that multimorbidity or several chronic conditions (such as arthritis, hypertension, diabetes and stroke) were associated with a higher risk of disability among older adults [24, 37, 38] and that the disabling effects of chronic diseases on disability weakened over time [8, 39]. One possible reason might be that earlier diagnosis and better medical services for multimorbidity decrease disability over time [2]. In addition, improvements in assistive devices and living environments might alleviate the limitations of chronic diseases on daily functions [31].

The main strength of this study was that the sample was drawn from a longitudinal and representative survey. Additionally, our study provided information on the prevalence of ADL and IADL disability among older Chinese adults over the past decade. However, there were several limitations in our study. First, physical examinations were not included, and self-reported measurements might lead to biases. Second, older people who were hospitalized or in nursing homes were not included, which may underestimate the prevalence of disability. Third, chronic diseases other than the 14 conditions were not included. Finally, we did not consider newly diagnosed chronic diseases in follow-up surveys in our analysis.

## Conclusion

This study provided evidence demonstrating an increasing trend in the prevalence of ADL and IADL disability among older Chinese adults from 2011 to 2018. Participants who were older, female, unmarried, living in rural areas and less educated were more likely to have ADL/IADL disability. The negative association between alcohol intake and IADL disability strengthened from 2011 to 2018. The disabling effects of the “arthritis/digestive diseases” pattern, “cardiometabolic diseases” pattern, and “high multimorbidity” pattern on ADL weakened over time. Considering the rising trends of disability in older adults, it is essential to improve long-term care services in China, especially for vulnerable groups (such as females and rural residents). Improving the prevention, diagnosis and treatment of multimorbidity would be helpful for reducing the risk of disability among older people. Building age-friendly communities and enhancing living conditions for older adults are also important strategies for preventing the rising trends in disability.

### Supplementary Information

Below is the link to the electronic supplementary material.

Supplementary file1 (DOCX 239 KB)

## Data Availability

The raw data is available on the website (http://charls.pku.edu.cn/en).
