# Supplementary material for: Trends in disability in activities of daily living and instrumental activities of daily living among Chinese older adults from 2011 to 2018
Source: Aging Clin Exp Res. 2024 Feb 7;36(1):27. doi: 10.1007/s40520-023-02690-7 (PMC10850014; doi:10.1007/s40520-023-02690-7)
Supplement: Supplementary file 1 — Supplementary file1 (DOCX 239 KB) [file 40520_2023_2690_MOESM1_ESM.docx]

**Supplementary Table 1** Comparison between models demonstrating best model fit for five latent classes

| Number of classes | BIC | aBIC | AIC | p for LMR |
| --- | --- | --- | --- | --- |
| 2 | 92158.6 | 92066.5 | 91951.4 | 0.00 |
| 3 | 91412.4 | 91272.5 | 91097.9 | 0.00 |
| 4 | 90938.0 | 90750.5 | 90516.4 | 0.00 |
| 5 | 90491.7 | 90256.5 | 89962.9 | 0.00 |
| 6 | 90266.2 | 89983.3 | 89630.2 | 0.00 |

**Supplementary Table 2** Trends in the prevalence of ADL or IADL disability for participants aged 60 and over, perfect match, 2011-2018

| **Variables** |  | **2011** | | **2013** | | **2015** | | **2018** | |  |
| --- | --- | --- | --- | --- | --- | --- | --- | --- | --- | --- |
|  |  | **n/N** | **Ref** | **n/N** | **OR (95%CI)** | **n/N** | **OR (95%CI)** | **n/N** | **OR (95%CI)** | **Ptrend†** |
| Total | ADL | 322/4623 | 1.00 | 344/4623 | 0.97(0.84-1.13) | 494/4623 | 1.37(1.19-1.58) | 649/4623 | 1.74(1.51-2.01) | <.001 |
|  | IADL | 738/4623 | 1.00 | 838/4623 | 1.09(0.99-1.21) | 985/4623 | 1.27(1.15-1.41) | 1342/4623 | 1.83(1.65-2.04) | <.001 |
| Age, years |  |  |  |  |  |  |  |  |  |  |
| 60-69 | ADL | 187/3253 | 1.00 | 170/2824 | 1.03(0.85-1.24) | 186/2333 | 1.40(1.15-1.69) | 119/1326 | 1.75(1.41-2.18) | <.001 |
|  | IADL | 447/3253 | 1.00 | 408/2824 | 1.03(0.91-1.18) | 375/2333 | 1.20(1.04-1.38) | 265/1326 | 1.69(1.44-1.99) | <.001 |
| 70-79 | ADL | 99/1166 | 1.00 | 130/1480 | 1.04(0.78-1.38) | 216/1808 | 1.53(1.17-1.99) | 316/2444 | 1.85(1.43-2.41) | <.001 |
|  | IADL | 224/1166 | 1.00 | 332/1480 | 1.30(1.07-1.57) | 427/1808 | 1.45(1.21-1.76) | 680/2444 | 2.12(1.77-2.55) | <.001 |
| ≥ 80 | ADL | 36/204 | 1.00 | 44/319 | 0.65(0.39-1.08) | 92/482 | 1.13(0.70-1.83) | 214/853 | 1.70(1.10-2.63) | <.001 |
|  | IADL | 67/204 | 1.00 | 98/319 | 0.98(0.68-1.39) | 183/482 | 1.43(0.99-2.05) | 397/853 | 2.33(1.64-3.29) | <.001 |
| Gender |  |  |  |  |  |  |  |  |  |  |
| Male | ADL | 132/2260 | 1.00 | 135/2260 | 0.95(0.76-1.18) | 188/2260 | 1.17(0.95-1.45) | 279/2260 | 1.75(1.40-2.17) | <.001 |
|  | IADL | 283/2260 | 1.00 | 339/2260 | 1.16(0.99-1.36) | 364/2260 | 1.17(0.99-1.37) | 547/2260 | 1.89(1.60-2.22) | <.001 |
| Female | ADL | 190/2363 | 1.00 | 209/2363 | 0.99(0.82-1.20) | 306/2363 | 1.53(1.27-1.85) | 370/2363 | 1.74(1.44-2.10) | <.001 |
|  | IADL | 455/2363 | 1.00 | 499/2363 | 1.05(0.92-1.19) | 621/2363 | 1.35(1.18-1.54) | 795/2363 | 1.80(1.56-2.06) | <.001 |
| Residence |  |  |  |  |  |  |  |  |  |  |
| Rural | ADL | 277/3699 | 1.00 | 277/3658 | 0.92(0.78-1.08) | 400/3658 | 1.30(1.11-1.52) | 528/3661 | 1.65(1.41-1.94) | <.001 |
|  | IADL | 645/3699 | 1.00 | 718/3658 | 1.08(0.97-1.20) | 833/3658 | 1.23(1.10-1.37) | 1123/3661 | 1.78(1.58-1.99) | <.001 |
| Urban | ADL | 45/923 | 1.00 | 67/964 | 1.34(0.95-1.88) | 94/964 | 1.84(1.31-2.60) | 121/962 | 2.33(1.67-3.24) | <.001 |
|  | IADL | 93/923 | 1.00 | 120/964 | 1.21(0.94-1.57) | 152/964 | 1.54(1.21-1.98) | 219/962 | 2.21(1.70-2.87) | <.001 |
| Education |  |  |  |  |  |  |  |  |  |  |
| No formal school | ADL | 213/2623 | 1.00 | 226/2619 | 0.96(0.80-1.16) | 321/2606 | 1.38(1.16-1.65) | 391/2450 | 1.69(1.41-2.02) | <.001 |
|  | IADL | 545/2623 | 1.00 | 601/2619 | 1.07(0.95-1.21) | 696/2606 | 1.24(1.09-1.41) | 879/2450 | 1.75(1.53-1.99) | <.001 |
| Elementary school | ADL | 67/1159 | 1.00 | 69/1158 | 0.95(0.69-1.30) | 111/1165 | 1.41(1.05-1.91) | 162/1231 | 1.94(1.43-2.64) | <.001 |
|  | IADL | 124/1159 | 1.00 | 157/1158 | 1.17(0.93-1.47) | 180/1165 | 1.29(1.02-1.62) | 286/1231 | 1.92(1.51-2.45) | <.001 |
| ≥ Middle school | ADL | 42/841 | 1.00 | 49/846 | 1.02(0.71-1.46) | 62/852 | 1.26(0.88-1.81) | 96/942 | 1.67(1.15-2.40) | .002 |
|  | IADL | 69/841 | 1.00 | 80/846 | 1.09(0.81-1.47) | 109/852 | 1.41(1.06-1.88) | 177/942 | 2.04(1.53-2.72) | <.001 |
| Marriage |  |  |  |  |  |  |  |  |  |  |
| Married | ADL | 259/3783 | 1.00 | 250/3660 | 0.93(0.79-1.09) | 355/3530 | 1.35(1.15-1.58) | 393/3259 | 1.61(1.36-1.89) | <.001 |
|  | IADL | 578/3783 | 1.00 | 636/3660 | 1.10(0.99-1.24) | 689/3530 | 1.23(1.09-1.39) | 859/3259 | 1.81(1.60-2.05) | <.001 |
| Unmarried | ADL | 63/840 | 1.00 | 94/963 | 1.14(0.83-1.56) | 139/1093 | 1.48(1.08-2.02) | 256/1364 | 2.16(1.59-2.94) | <.001 |
|  | IADL | 160/840 | 1.00 | 202/963 | 1.05(0.84-1.30) | 296/1093 | 1.37(1.11-1.69) | 483/1364 | 1.85(1.49-2.30) | <.001 |
| Alcohol intake | |  |  |  |  |  |  |  |  |  |
| > once/month | ADL | 63/1124 | 1.00 | 44/1134 | 0.67(0.45-0.98) | 73/1106 | 1.05(0.74-1.49) | 76/1038 | 1.15(0.78-1.70) | .149 |
|  | IADL | 135/1124 | 1.00 | 133/1134 | 0.97(0.75-1.24) | 138/1106 | 0.97(0.75-1.24) | 176/1038 | 1.43(1.12-1.83) | .002 |
| ≤ once/month or never | ADL | 259/3497 | 1.00 | 298/3483 | 1.03(0.88-1.21) | 420/3514 | 1.44(1.23-1.68) | 573/3585 | 1.84(1.58-2.15) | <.001 |
|  | IADL | 602/3497 | 1.00 | 703/3483 | 1.12(1.00-1.25) | 846/3514 | 1.34(1.19-1.50) | 1166/3585 | 1.91(1.69-2.14) | <.001 |
| Smoking |  |  |  |  |  |  |  |  |  |  |
| Ever smoking | ADL | 122/1887 | 1.00 | 140/2081 | 0.95(0.77-1.18) | 213/2170 | 1.31(1.07-1.61) | 287/2111 | 1.81(1.46-2.26) | <.001 |
|  | IADL | 271/1887 | 1.00 | 343/2081 | 1.08(0.92-1.26) | 401/2170 | 1.16(0.99-1.36) | 561/2111 | 1.81(1.53-2.13) | <.001 |
| Never smoking | ADL | 200/2735 | 1.00 | 204/2541 | 0.99(0.81-1.20) | 281/2453 | 1.42(1.18-1.72) | 362/2512 | 1.68(1.40-2.03) | <.001 |
|  | IADL | 466/2735 | 1.00 | 494/2541 | 1.09(0.96-1.24) | 584/2453 | 1.34(1.17-1.54) | 781/2512 | 1.84(1.60-2.12) | <.001 |

*Notes*: ADL=activities of daily living; IADL=instrumental activities of daily living; OR=odds ratio; CI=confidence interval. Adjusting for age, gender, residence, education, marital status, ever smoking, alcohol intake, and multimorbidity

**Supplementary Table 3** Average annual changes at 95% Confidence Interval in the odds ratio of ADL/IADL disability, perfect match, 2011-2018

|  |  | Average Annual Change (95%CI), % | | | |  |
| --- | --- | --- | --- | --- | --- | --- |
| **Variables** |  | **Model 1** | **Model 2** | **Model 3** | **Model 4** | **P** |
| Total | ADL | 12.1(10.5-13.7) | 9.1(7.3-10.9) | 8.9(7.1-10.7) | 9.0(7.1-11.0) | <.001 |
|  | IADL | 11.3(10.2-12.5) | 8.9(7.5-10.2) | 8.7(7.4-10.1) | 9.1(7.6-10.6) | <.001 |
| Age (years) | |  |  |  |  |  |
| 60-69 | ADL | 8.6(5.9-11.4) | 8.5(5.7-11.2) | 8.3(5.5-11.0) | 8.7(5.6-11.7) | <.001 |
|  | IADL | 6.7(4.7-8.8) | 6.9(4.8-9.0) | 6.8(4.7-9.0) | 7.3(5.0-9.7) | <.001 |
| 70-79 | ADL | 8.9(6.0-11.8) | 9.5(6.5-12.4) | 9.5(6.5-12.4) | 9.7(6.6-12.9) | <.001 |
|  | IADL | 8.7(6.6-10.8) | 10.0(7.8-12.2) | 9.9(7.7-12.1) | 10.5(8.2-12.8) | <.001 |
| ≥80 | ADL | 13.8(8.8-18.8) | 14.1(9.1-19.2) | 13.6(8.5-18.8) | 12.4(7.0-17.8) | <.001 |
|  | IADL | 14.4(10.6-18.3) | 15.4(11.5-19.4) | 15.0(11.0-19.1) | 14.3(10.0-18.5) | <.001 |
| Gender | |  |  |  |  |  |
| Male | ADL | 13.1(10.6-15.5) | 9.8(7.0-12.6) | 9.4(6.5-12.2) | 9.1(6.1-12.1) | <.001 |
|  | IADL | 11.7(9.9-13.5) | 9.0(7.0-11.1) | 8.6(6.5-10.7) | 9.2(7.0-11.5) | <.001 |
| Female | ADL | 11.3(9.2-13.4) | 8.5(6.2-10.8) | 8.5(6.2-10.8) | 8.7(6.1-11.2) | <.001 |
|  | IADL | 11.3(9.7-12.9) | 8.7(7.0-10.5) | 8.8(7.0-10.6) | 9.0(7.1-10.9) | <.001 |
| Residence | |  |  |  |  |  |
| Rural | ADL | 11.7(9.9-13.5) | 8.6(6.6-10.6) | 8.4(6.4-10.4) | 8.5(6.4-10.7) | <.001 |
|  | IADL | 11.0(9.7-12.3) | 8.4(6.9-9.9) | 8.3(6.8-9.8) | 8.7(7.1-10.3) | <.001 |
| Urban | ADL | 14.2(10.7-17.8) | 11.8(7.9-15.8) | 12.0(8.1-16.0) | 11.6(7.2-15.9) | <.001 |
|  | IADL | 14.1(11.3-16.9) | 11.2(7.9-14.5) | 11.3(8.0-14.5) | 11.5(8.0-15.0) | <.001 |
| Education | |  |  |  |  |  |
| No formal school | ADL | 12.0(9.9-14.0) | 8.5(6.3-10.8) | 8.4(6.1-10.6) | 8.6(6.1-11.0) | <.001 |
|  | IADL | 11.1(9.5-12.6) | 8.0(6.3-9.7) | 7.9(6.2-9.6) | 8.4(6.5-10.2) | <.001 |
| Elementary school | ADL | 14.0(10.6-17.3) | 11.4(7.5-15.3) | 11.2(7.3-15.1) | 10.9(6.7-15.2) | <.001 |
|  | IADL | 13.1(10.5-15.7) | 9.8(6.9-12.7) | 9.5(6.5-12.5) | 9.5(6.2-12.7) | <.001 |
| Middle school or above | ADL | 11.1(6.8-15.4) | 7.9(3.2-12.5) | 7.7(3.0-12.4) | 8.0(3.0-13.1) | .002 |
|  | IADL | 13.9(10.6-17.2) | 10.6(6.9-14.2) | 10.6(6.9-14.2) | 11.0(7.1-14.8) | <.001 |
| Marriage | |  |  |  |  |  |
| Married | ADL | 10.4(8.5-12.3) | 7.7(5.6-9.8) | 7.5(5.4-9.7) | 7.9(5.6-10.2) | <.001 |
|  | IADL | 10.1(8.7-11.5) | 8.1(6.5-9.7) | 8.0(6.3-9.6) | 8.7(7.0-10.4) | <.001 |
| Unmarried | ADL | 15.2(11.9-18.4) | 12.4(8.9-16.0) | 12.1(8.6-15.7) | 11.9(8.0-15.7) | <.001 |
|  | IADL | 12.9(10.5-15.3) | 10.4(7.7-13.0) | 10.2(7.5-12.9) | 9.8(6.9-12.6) | <.001 |

*Notes*: CI=Confidence Interval. Model 1 was univariate; Model 2 was controlled for age, gender, residence, education and marital status; Model 3 was additionally controlled for lifestyle factors (ever smoking and alcohol intake); and Model 4 was further controlled for multimorbidity.

**Supplementary Table 4** Trends in the associations of unhealthy behaviors and chronic diseases on ADL/IADL disability, perfect match, 2011-2018

| **Variables** |  | **2011 (n = 4783)** | | **2013 (n = 4783)** | | **2015 (n = 4783)** | | **2018 (n = 4783)** | | Interaction  effect | Ptrend* |
| --- | --- | --- | --- | --- | --- | --- | --- | --- | --- | --- | --- |
|  |  | n/N | OR (95%CI) | n/N | OR (95%CI) | n/N | OR (95%CI) | n/N | OR (95%CI) |  |  |
| Alcohol intake | ADL | 63/1124 | 0.89(0.64-1.24) | 44/1134 | 0.56(0.39-0.80) | 73/1106 | 0.69(0.51-0.94) | 76/1038 | 0.50(0.38-0.66) | -.028 | .271 |
|  | IADL | 135/1124 | 0.80(0.63-1.01) | 133/1134 | 0.66(0.53-0.83) | 138/1106 | 0.62(0.49-0.78) | 176/1038 | 0.54(0.44-0.66) | -.035 | .051 |
| Ever smoking | ADL | 122/1887 | 1.12(0.81-1.54) | 140/2081 | 1.13(0.83-1.55) | 213/2170 | 1.30(0.97-1.72) | 287/2111 | 1.30(1.03-1.66) | .023 | .222 |
|  | IADL | 271/1887 | 1.19(0.95-1.50) | 343/2081 | 1.06(0.85-1.32) | 401/2170 | 1.18(0.95-1.48) | 561/2111 | 1.19(0.99-1.45) | .002 | .911 |
| Multimorbidity pattern | | | |  |  |  |  |  |  |  |  |
| No chronic diseases | ADL | 30/1192 | 1.00(ref) | 64/1410 | 1.00(ref) | 97/1580 | 1.00(ref) | 138/1589 | 1.00(ref) | — | — |
|  | IADL | 116/1192 | 1.00(ref) | 187/1410 | 1.00(ref) | 245/1580 | 1.00(ref) | 343/182 | 1.00(ref) | — | — |
| Arthritis/digestive | ADL | 114/1547 | 2.25(1.43-3.54) | 93/1425 | 0.82(0.56-1.20) | 145/1301 | 1.46(1.04-2.07) | 182/1296 | 1.38(1.06-1.80) | -.042 | .109 |
|  | IADL | 244/1547 | 1.30(0.99-1.70) | 264/1425 | 1.08(0.84-1.37) | 280/1301 | 1.24(0.97-1.58) | 368/1296 | 1.21(0.99-1.47) | -.021 | .244 |
| Respiratory | ADL | 26/383 | 1.84(1.02-3.32) | 33/366 | 1.04(0.64-1.70) | 51/359 | 1.93(1.25-2.99) | 75/357 | 1.94(1.37-2.74) | .027 | .453 |
|  | IADL | 68/383 | 1.44(1.00-2.08) | 86/366 | 1.37(0.99-1.90) | 92/359 | 1.55(1.11-2.16) | 135/357 | 2.01(1.52-2.65) | .037 | .148 |
| Cardiometabolic | ADL | 102/1141 | 2.78(1.75-4.43) | 107/1088 | 1.16(0.79-1.70) | 136/1056 | 1.70(1.20-2.41) | 184/1055 | 1.70(1.30-2.23) | -.042 | .115 |
|  | IADL | 197/1141 | 1.52(1.14-2.02) | 208/1088 | 1.09(0.84-1.41) | 253/1056 | 1.41(1.10-1.82) | 353/1055 | 1.54(1.25-1.89) | .006 | .741 |
| High multimorbidity | ADL | 31/184 | 4.90(2.67-9.01) | 30/184 | 1.80(1.06-3.05) | 38/182 | 2.84(1.74-4.63) | 42/182 | 2.13(1.39-3.28) | -.090 | .022 |
|  | IADL | 60/184 | 3.16(2.06-4.85) | 54/184 | 1.76(1.18-2.63) | 69/182 | 2.73(1.85-4.04) | 81/182 | 2.30(1.61-3.28) | -.037 | .247 |
| Multimorbidity | |  |  |  |  |  |  |  |  |  |  |
| No | ADL | 106/2521 | 1.00(ref) | 80/2150 | 1.00(ref) | 111/1877 | 1.00(ref) | 191/2129 | 1.00(ref) | — | — |
|  | IADL | 287/2521 | 1.00(ref) | 263/2150 | 1.00(ref) | 271/1877 | 1.00(ref) | 471/2129 | 1.00(ref) | — | — |
| Yes | ADL | 213/2032 | 1.82(1.37-2.43) | 255/2393 | 2.86(2.07-3.95) | 326/2251 | 1.87(1.41-2.48) | 458/2494 | 1.74(1.39-2.17) | -.040 | .037 |
|  | IADL | 434/2032 | 1.67(1.36-2.06) | 549/2393 | 2.00(1.64-2.46) | 591/2251 | 1.66(1.35-2.04) | 871/2494 | 1.55(1.31-1.83) | -.011 | .448 |

*Notes*: ADL=activities of daily living; IADL=instrumental activities of daily living; OR=odds ratio; CI=confidence interval. Adjusting for age, gender, residence, education, marital status, ever smoking and alcohol intake in the table.


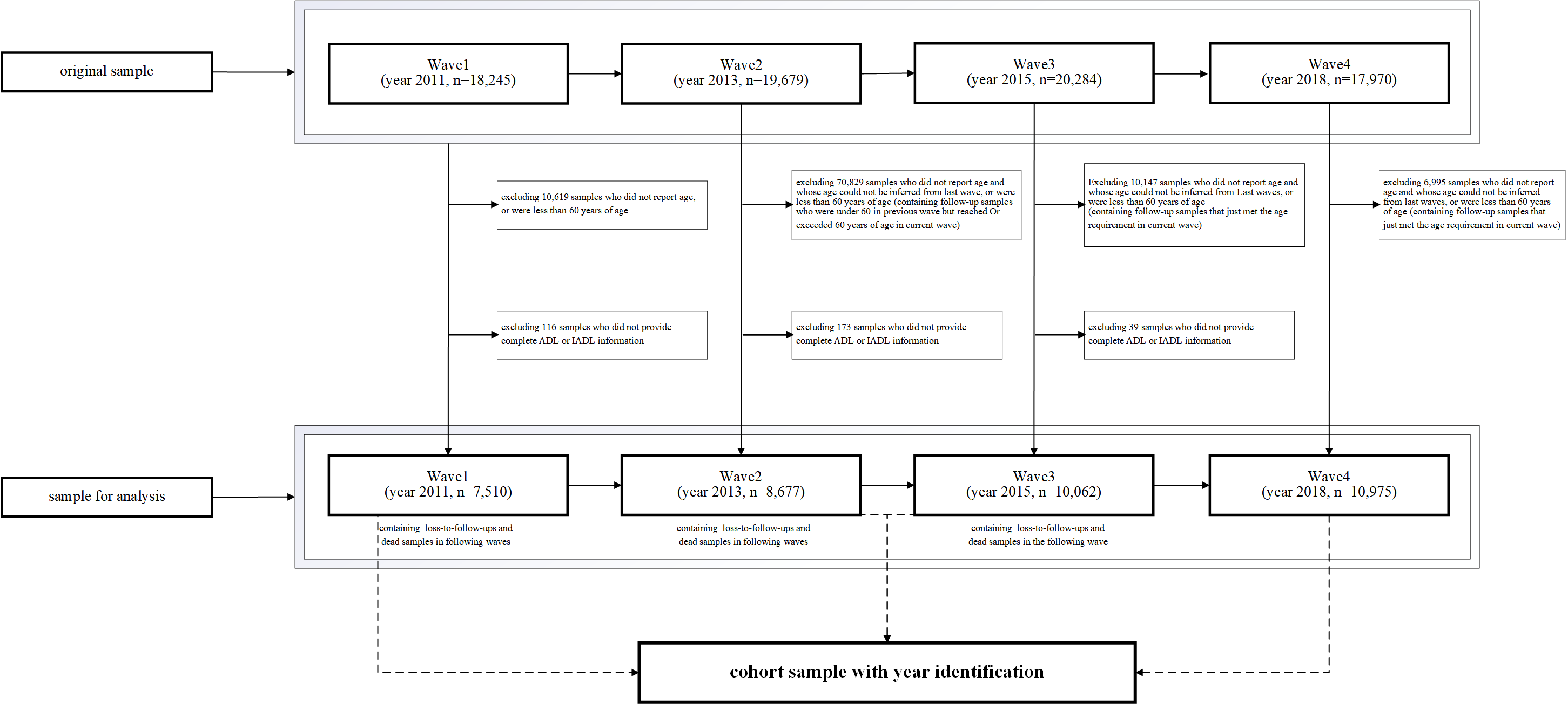


**Supplementary Figure 1** Flowchart of cohort sample


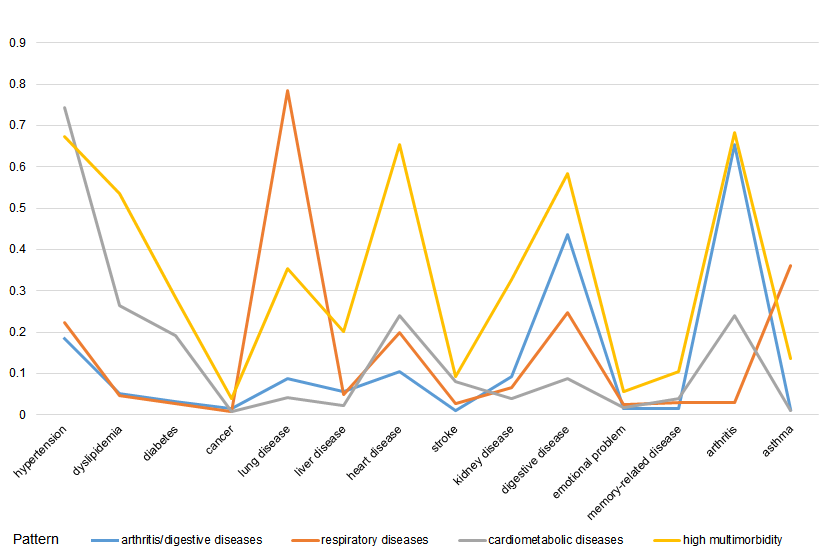


**Supplementary Figure 2** Conditional prevalence of chronic conditions by patterns
